# Occupational health, cognitive disorders and occupational neuropsychology

**DOI:** 10.1590/S1980-57642012DN06040002

**Published:** 2012

**Authors:** Leonardo Caixeta, George Martins Ney da Silva Júnior, Victor de Melo Caixeta, Cláudio Henrique Ribeiro Reimer, Paulo Verlaine Borges e Azevedo

**Affiliations:** 1 MD, PhD, Associate Professor of Neuropsychiatry, School of Medicine of Federal University of Goiás (UFG). Coordinator, Cognitive and Behavioral Neurology Unit, Hospital das Clínicas - UFG, Goiânia GO, Brazil.; 2 MD, Behavioral and Cognitive Neurology Unit, Hospital das Clínicas, Federal University of Goiás, Goiânia GO, Brazil.; 3 MD, PhD, Professor of Psychiatry, Pontifical Catholic University of Goiás (PUC-GO), Goiânia GO, Brazil.

**Keywords:** occupational health, cognitive disorders, neuropsychology, executive functions, burnout

## Abstract

Work can be an important etiologic factor in the genesis of some mental disorders including cognitive disability. Occupational neuropsychology constitutes an intriguing new but neglected area of research and clinical practice which deals with the neurocognitive consequences of the work environment and work habits. Neuropsychological knowledge is fundamental to understand cognitive requirements of work competence. Work can impact sleep patterns and mental energy, which in turn can cause neuropsychological symptoms. This report presents relevant evidence to illustrate the relationship between work and cognitive dysfunction.

## Abstract (pt)

O trabalho pode ser um importante fator etiológico na gênese de algumas doenças mentais, incluindo transtornos cognitivos. A neuropsicologia ocupacional constitui uma intrigante, mas negligenciada nova área de pesquisa e atuação clínica, que lida com as consequências neurocognitivas do ambiente e dos hábitos de trabalho. O conhecimento neuropsicológico é fundamental para se entender os pressupostos cognitivos da competência no trabalho. O trabalho pode afetar os padrões de sono e de energia mental, que por sua vez podem causar sintomas neuropsicológicos. Foi realizada uma apresentação de evidências relevantes para ilustrar a relação entre trabalho e disfunção cognitiva.

## INTRODUCTION

Mental health problems have contributed increasingly to absenteeism at work and long-term disability,^1^ but the reverse can also occur: work can be an important etiologic factor in the genesis of some mental disorders including cognitive disability.^2^

Neuropsychology, covers a wide field of expertise and accumulated knowledge, can be subdivided into several subareas, including occupational neuropsychology which deals with the neurocognitive consequences of the work environment. Occupational neuropsychology encompasses and supports several medical specialties, including occupational psychiatry whose recent revival is due to the increasing number of mental disorders in work activities and new laws protecting employee health.^3,4^ In this latest approach, both occupational neuropsychology and occupational psychiatry are faced with a lack of concepts and current approaches, prompting us to study this issue.

The high incidence of mental disorders among workers - around 30% for minor mental disorders and 5%-10% for severe mental disorders^5^ coupled with the granting of social security benefits for those insured under Social Security ("psychoneuroses" ranks first among the causes of temporary disability and second and third among the causes of permanent disability6) show the importance of occupational psychiatry and occupational neuropsychology.^3,4^ The weight of statistics, plus the efforts of researchers, has led to legal recognition of the relationship between mental health and work, by Decree No. 3.048/99, from the Brazilian Ministry of Social Security, which deals with Work-Related Mental Disorders.

In this brief review, we present relevant evidence that illustrates and highlights the relationship between work and mental (including cognitive) function and dysfunction, contributing to structure knowledge in this field. Occupational neuropsychology is presented according to its most characteristic and structural aspects. In the present aggregated review of the literature, a search of the databases SciELO and MEDLINE was conducted using the key words "occupational health" "and" "cognition". The most representative articles on the subject were selected, dedicating special attention to the contributions of internationally renowned investigators. Reference books that were pertinent under the framework of this review article were also consulted.

## WORK AND MENTAL HEALTH

The knowledge that work can cause illness is ancient. The very origin of the meaning of "work" alludes to its dual status as an activity that involves suffering and achievement, since the Latin word *trapalium* ("work") means a three-piece instrument which was used both as an agricultural tool and an instrument of torture. The theoretical understanding of the underlying health of workers is based on a body of knowledge from different disciplines such as Social Medicine, Public Health, Internal Medicine, Labor Medicine, Sociology, Social Epidemiology and Engineering among many others. This understanding, allied with the knowledge of the worker at their workstation and experiences of burden, represents a new approach to understanding the relationship between health and work and proposes a new practice of healthcare professionals and intervention in the workplace.^7^ Occupational diseases are easily recognized as an effect of en vironmental hazards to which workers are exposed, whereas the relationship between mental disorders and work are less recognized, i.e., the so-called psychosocial risks that include phenomena of a neurophysiological, cognitive, psychological, social and organizational nature.^7,8^

The multiple and complex aspects involved in the workplace, work environment, economic issues, work relationships and workers health, including mental health, define the need for government involvement in the provision of laws, economic incentives - policies, in short - to attenuate the effects of work distress on workers' mental health, as reported by some researchers.^9^

## MENTAL CAPACITY AND WORK SUCCESS

There are several factors affecting the ability to work. Among these are the influence of age,^9-11^ shift work^12^ and particularly the presence of psychiatric symptoms.^13^ The success of labor is typically conceptualized in two main dimensions: an extrinsic dimension (objective) and an intrinsic dimension (subjective).^14^ The extrinsic dimension refers to measurable variables, such as salary, promotions, career and quality of work scrutinized by others. The intrinsic dimension refers to the self-assessment of levels of satisfaction related to job performance.^15^ This evaluation process is intrinsically related to the concept of work ability.^16^ According to some authors^17^ the "ability to work" is defined as the self-assessment the worker makes regarding their well-being now and in the near future and the ability to ensure their work in terms of its requirements, health and available psychological resources.

## EXECUTIVE FUNCTION AND WORK

Over recent years in the neuropsychological field, there has been growing interest in the concept and assessment of executive cognitive functions and how these functions relate to health and adaptive behaviors, indispensable for personal, social and professional well-being.^18^ The concept includes a set of higher-order cognitive functions, which are instrumental in self-regulation of human behavior,^19^ such as abstract reasoning and problem solving;^20^ planning, organizing and self-monitoring of actions that target a specific goal;^21^ and inhibition of irrelevant behaviors as well as mental flexibility.^19^ In this sense, a lack of integrity or impairment of executive functions may impair effective work performance, including the ability to work.^18^

The relationship between work ability and executive cognitive functions, according to some authors,^15^ may be mediated or moderated by certain personality traits. According to other authors,^22^ high levels of neuroticism have a negative influence on the levels of decision-making and consequent injury at work, while high levels of extroversion and conscientiousness positively influence both executive functioning and the ability to work. The characterization of these variables, and understanding how they interrelate, is crucial for the implementation of techniques and preventive strategies to promote health among workers, contributing to an improvement in their ability to work and their executive functioning, favoring the development of a better quality of life.

## TRIGGERING FACTORS IN THE PROCESS OF NEUROPSYCHOLOGICAL DYSFUNCTION AT WORK

Some authors^23^ have reported elements that act as triggers of the process of cognitive malfunctioning at work, namely: lack of time control at work (long hours, night or alternating shifts, etc.); requirement for high levels of attention and concentration to perform tasks; occupational exposure (especially to heavy metals and solvents). However, the establishment of a causal link with work is complex, since the morbidity process has an element of specificity and involves multiple aspects of the subject.

## WORK, SLEEP DEPRIVATION AND COGNITIVE PERFORMANCE

Reduced sleep is a frequent effect of suboptimal work schedules or shift work. On the other hand, sleep deprivation results in significant impairments in cognitive and motor performance which increase for instance the risk of motor vehicle crashes and work-related injuries and fatal accidents.^24^ Empirical data from a study^25^ correlating accidents among interns and sleep deprivation on duties and night-shift work determined that each extended work shift scheduled during a month increased the monthly risk of a motor vehicle crash by 9.1 percent (95 percent confidence interval, 3.4 to 14.7 percent) and increased the monthly risk of a crash during the commute from work by 16.2 percent (95 percent confidence interval, 7.8 to 24.7 percent). On months in which interns worked five or more extended shifts, the risk of falling asleep at the wheel while driving or while stationary in traffic was significantly increased (odds ratios, 2.39 [95 percent confidence interval, 2.31 to 2.46] and 3.69 [95 percent confidence interval, 3.60 to 3.77], respectively).

Sleep and cognition are temporally regulated by a homeostatic process generating pressure for sleep as a function of sleep/wake history, and a circadian process generating pressure for wakefulness as a function of time of day. Under normal nocturnal sleep conditions, these two processes are aligned in such a manner as to provide optimal daytime performance and consolidated nighttime sleep. However, under conditions of sleep deprivation, shift work or trans-meridian travel, the two processes become misaligned, resulting in fatigue and cognitive deficits.^25^

Some authors^26^ investigated simple and high-order cognitive performance change after one night of sleep deprivation (SD) and recovery after 7 h of normal recovery sleep opportunity over three recovery days. According to these authors, performance on simple tasks such as addition or short-term memory, was not reduced after SD and was poorest on the baseline day, improving gradually thereafter. High-order cognitive performances were at their lowest on the post-vigil day and needed 2 recovery sleep opportunities to return to baseline levels.

Other authors report that short-term memory appears to decline after day and overnight shifts, based on data collected from a study involving attending emergency physicians before and after day and overnight shifts.^27^ Along the same line of investigation, Rollinson et al. studied interns working nights in an emergency department and showed a significant reduction in visual memory capacity during the night shift.^28^

## WORK, FATIGUE AND COGNITIVE PERFORMANCE

For over a century, it has been recognized that long working hours can lead to a greater perception of fatigue by workers and to a reduction in worker efficiency. The role of fatigue in worker health and safety, measured in terms of accident and absenteeism rates, as a result of longer workdays has also been examined, and some authors have explored the effects of acute or short-term fatigue (i.e., over a prolonged workday) on job performance.^27-30^ Fatigue resulting from several days of a long work schedule (e.g. a compressed work week shift, sustained operations work, hospital on-call service by physicians and nurses) affects behavioral or psychological test performances.^29^

Prolonged cognitive load associated with some professions can cause fatigue, which, in turn, can dramatically impact cognitive functions by decreasing mental energy.^30^ Mental fatigue is reflected by a failure to complete mental tasks that require self-motivation and internal cues, in the absence of demonstrable cognitive failure or motor weakness. Lieberman^31^ suggests use of cognitive tests that assess vigilance, ability to sustain attention, and choice reaction time for the assessment of mental energy and fatigue. Some authors^32^ have shown that compared to a non-fatigued group, fatigued participants displayed more perseveration on the *Wisconsin Card Sorting Test* and showed prolonged planning time on the *Tower of London* task. Fatigue did not however, affect performance on a simple memory task. These authors concluded by highlighting compromised executive control under fatigue, which may explain the typical errors and sub-optimal performance often found in fatigued individuals.^32^

Burnout is an important concept relevant to the present issue. This refers to a work syndrome first described in physicians characterized as a unique affective multidimensional response to stress, the core components of which are emotional exhaustion, physical fatigue, and cognitive weariness.^33^ The phenomenon of burnout has a chronic nature and exhibits remarkable stability over time regardless of sample makeup, cultural context, and length of time of follow-up survey.^34^ Oosterholt et al.^35^ suggested that burnout leads to permanent cognitive deficits, that subjective burnout complaints reduce faster than deficits in cognitive test performance, or that cognitive deficits are a cause rather than a consequence of burnout.

At least two available instruments have been designed for assessing work burnout: the Copenhagen Burnout Inventory^36^ (CBI) and the Maslach Burnout Inventory.^37^ Despite the importance of the burnout phenomenon, few systematic investigation assessing work as a cause of burnout are available, although more attention has been dedicated to burnout as a consequence of disease. Systematic approaches to burnout as a consequence of work that go beyond the care of patients can shed light on the condition and help its prevention.

International commercial airline pilots may experience heightened fatigue due to irregular sleep schedules, long duty days, night flying, and multiple time zone changes. Some authors^36^ have investigated the relationship among commercial airline pilots' amount of sleep, subjective fatigue, and sustained attention before and after international flights using a 5 min PalmPilot-based psychomotor vigilance task and self-rating of level of fatigue using the Samn-Perelli Fatigue Checklist. A significant main effect of stage of flight was found for self-rated fatigue and mean response speed.

Proctor et al.^29^ examined whether increased overtime work among automotive workers predicted impairment in cognitive performance in the domains of attention and executive function. These researchers found that overtime work affects cognitive function as measured by neuropsychological testing, with impairments observed in tasks requiring attention and executive function skills.

**Conclusions.** Occupational neuropsychology constitutes an intriguing new but neglected area of research and clinical practice which deals with the neurocognitive consequences of the work environment and work habits. Neuropsychological knowledge is fundamental to understand cognitive requirements of work competence. Work can impact sleep patterns and mental energy, which in turn can cause neuropsychological symptoms. Recognition that increased overtime work affects cognitive function in certain functional areas and, by extension, may affect worker performance, has important implications for the health and safety of workers as well as for the economic management of the workplace. In addition, better understanding of neuropsychological implications of these work dysfunctions, and their relationships with work ability and workers' mental health, will allow assessment and preventive programs to be devised as well as more specific and efficient therapeutic tools for managing the mental distress triggered, exacerbated or caused by work.
